# GABAergic inhibition is weakened or converted into excitation in the oxytocin and vasopressin neurons of the lactating rat

**DOI:** 10.1186/s13041-015-0123-0

**Published:** 2015-05-28

**Authors:** Seung Won Lee, Young-Beom Kim, Jeong Sook Kim, Woong Bin Kim, Yoon Sik Kim, Hee Chul Han, Christopher S. Colwell, Young-Wuk Cho, Yang In Kim

**Affiliations:** Department of Physiology, Korea University College of Medicine, 126-1 Anam-dong 5-ga, Seoul, 136-705 Republic of Korea; Neuroscience Research Institute, Korea University College of Medicine, Seoul, 136-705 Republic of Korea; Department of Physiology, Biomedical Science Institute & Medical Research Center, School of Medicine, Kyung Hee University, Seoul, 130-701 Republic of Korea; Department of Psychiatry & Biobehavioral Sciences, University of California-Los Angeles, Los Angeles, CA 90024 USA

**Keywords:** Electrophysiology, GABA, KCC2, Lactation, NKCC1, Oxytocin, Vasopressin

## Abstract

**Background:**

Increased secretion of oxytocin and arginine vasopressin (AVP) from hypothalamic magnocellular neurosecretory cells (MNCs) is a key physiological response to lactation. In the current study, we sought to test the hypothesis that the GABA_A_ receptor-mediated inhibition of MNCs is altered in lactating rats.

**Results:**

Gramicidin-perforated recordings in the rat supraoptic nucleus (SON) slices revealed that the reversal potential of GABA_A_ receptor-mediated response (E_GABA_) of MNCs was significantly depolarized in the lactating rats as compared to virgin animals. The depolarizing E_GABA_ shift was much larger in rats in third, than first, lactation such that GABA exerted an excitatory, instead of inhibitory, effect in most of the MNCs of these multiparous rats. Immunohistochemical analyses confirmed that GABAergic excitation was found in both AVP and oxytocin neurons within the MNC population. Pharmacological experiments indicated that the up-regulation of the Cl^−^ importer Na^+^-K^+^-2Cl^−^ cotransporter isotype 1 and the down-regulation of the Cl^−^ extruder K^+^-Cl^−^ cotransporter isotype 2 were responsible for the depolarizing shift of E_GABA_ and the resultant emergence of GABAergic excitation in the MNCs of the multiparous rats.

**Conclusion:**

We conclude that, in primiparous rats, the GABAergic inhibition of MNCs is weakened during the period of lactation while, in multiparous females, GABA becomes excitatory in a majority of the cells. This reproductive experience-dependent alteration of GABAergic transmission may help to increase the secretion of oxytocin and AVP during the period of lactation.

## Introduction

GABA, a major neurotransmitter in the mammalian central nervous system (CNS), acts through ionotropic GABA_A_/GABA_C_ or metabotropic GABA_B_ receptors [1]. When GABA opens GABA_A_ channel in a CNS neuron, chloride ion (Cl^−^) flows into the cell through this channel down its electrochemical gradient, inhibiting the neuron’s electrical activity [2]. However, there are conditions in which the electrochemical gradient for Cl^−^ is set toward the extracellular side due to high intracellular Cl^−^ concentration ([Cl^−^]_i_). In these conditions, Cl^−^ efflux rather than influx occurs when GABA_A_ channel is activated which can depolarize the membrane potential and even generate action potentials. For example, in pathological conditions such as hyperalgesia and hypertension, GABA exerts an excitatory effect in some neurons as a part of the etiology [3, 4]. Moreover, in immature cortical neurons whose Cl^−^-extruding capability is low [5], GABA can depolarize the membrane potential to elicit action potentials [6]. This GABA-mediated excitation is thought to be important for various events occurring in CNS development including neural circuit formation. Lastly, in certain normal mature CNS neurons, GABA can elicit depolarizing/excitatory responses, but the physiological significance of this unusual GABAergic effect is unclear [7–11].

During the last several years, we have been interested in the question of whether GABA_A_ receptor-mediated transmission in mature CNS neurons can switch between inhibition and excitation in response to physiological need. We have found that in the magnocellular neurosecretory cells (MNCs) of the paraventricular (PVN) and supraoptic nuclei (SON) of the rat, GABAergic inhibition is converted into excitation in a reversible fashion in response to chronic hyperosmotic stress given by 2 % NaCl as drinking solution. This switch enhances the secretion of the antidiuretic hormone arginine-vasopressin (AVP) and the natriuretic hormone oxytocin from these neurons [12]. In the current study, we investigated whether the GABAergic inhibition of MNCs is weakened or converted into excitation during the period of lactation. We envisioned that, if indeed such a change occurs, the secretion of oxytocin, a neurohormone with milk-ejecting function in lactating mammals [13], and AVP, another neurohormone which helps to prevent the mother from being dehydrated during lactation and thus maintain milk yield [14–16], would significantly increase, considering that the output of MNCs is regulated by the dense GABAergic innervation [17]. In testing this hypothesis, we utilized female rats in four conditions: 1) virgin, 2) lactating after giving the first successful birth (Lac1), 3) lactating after giving birth 3 times (Lac3) and 4) in dry period after giving birth 3 times (Dry3). We reasoned that the change in the strength or polarity of GABAergic transmission, if occurs, would be more marked in the Lac3, than Lac1, rats since milk yield and maternal behavior, both of which rely on oxytocin and AVP ([18–23], but also see [24, 25]), are known to be enhanced in multiparous mammals compared to primiparous ones [26–29].

## Results

### Milk yield was greater in multiparous rats

**Fig. 1:**
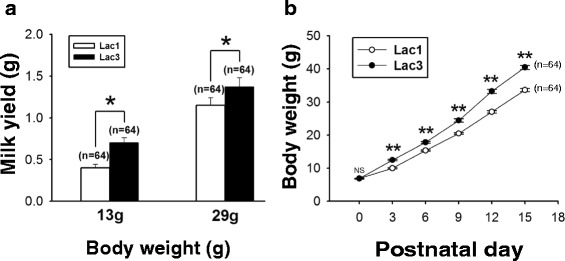
Difference between Lac1 and Lac3 rats in milk yield and pup growth. (**a**) Bar charts showing difference between Lac1 and Lac3 rats in milk yield at two different time points when the average body weights of their pups were 13 g and 29 g. The milk yield is defined in the Results section. Data from 128 pups nursed by 8 Lac1 and 8 Lac3 dams. Each dam nursed a litter of 8 pups for 1 h. (**b**) Line graphs showing the difference between the pups of Lac1 and Lac3 rats in their weight gain over the period of PND 0–15 (*: *p* < 0.05; **: *p* < 0.001; Student’s *t*-test). n: the number of pups. In this and the rest of the figures, values are shown as means (± SEM)

To determine if milk production is larger in Lac3 than Lac1 rats, we compared the milk yields of these groups of rats at two different time points, i.e., when the average body weights of their pups were 13 g and 29 g. To measure the milk yield, we let the dam nurse a litter of 8 pups for 1 h. The milk yield was defined as the difference in body weight of the pup measured 4 h before and immediately after the nursing. Milk yield was significantly greater in Lac3, than Lac1, rats at both time points (Fig. 1a). As an indirect index of milk production, the body weight of pup was also monitored over postnatal day (PND) 0–15. The body weight of Lac3 pups was significantly greater than that of Lac1 pups at PND 3–15 (Fig. 1b). Collectively, these results indicated that milk yield was greater in multiparous rats.

### GABA was excitatory in most of the MNCs of rats in third lactation

**Fig. 2:**
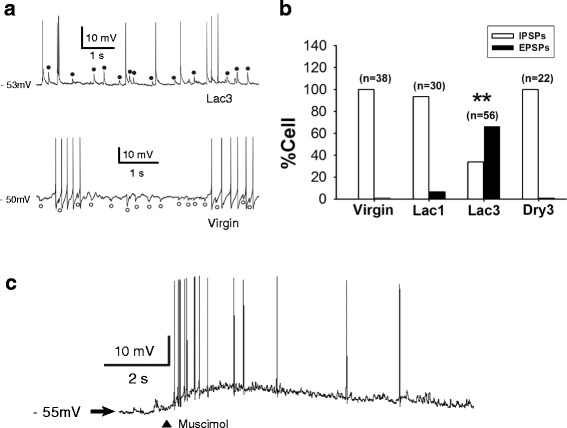
Conversion of GABA_A_ receptor-mediated inhibition to excitation in the MNCs of lactating rats. (**a**) Spontaneously occurring fast GABAergic excitatory postsynaptic potentials (EPSPs, ●) and inhibitory postsynaptic potentials (IPSPs, ○) recorded in the presence of AP-5 (100 μM) and DNQX (20 μM) from the MNCs of Lac3 (*upper trace*) and virgin rats (*lower trace*). Note the action potentials that arise from the GABAergic EPSPs (*upper trace*). (**b**) Proportions of the MNCs having GABAergic EPSPs and IPSPs in different groups of rats. (**: *p* < 0.001, Chi-square test). n: the number of cells examined. These groups of rats were not significantly different from each other in the input resistance of MNC (Virgin: 308 ± 12 MΩ, n = 38; Lac1: 320 ± 15 MΩ, n = 30; Lac3: 303 ± 12 MΩ, n = 56; Dry3: 313 ± 12 MΩ, n = 22). (**c**) Depolarization and associated action potentials elicited by focal application of the GABA_A_ agonist muscimol (10 μM; 10 ms) in an MNC of Lac3 rat

Next, we examined fast GABAergic postsynaptic potentials (PSPs) occurring spontaneously in the MNCs sampled in the SON slices of the Virgin, Lac1, Lac3 and Dry3 groups of rats. These PSPs were recorded in the presence of DL-2-amino-5-phosphonopentanoic acid (AP-5, 100 μM) and 6,7-dinitroquinoxaline-2,3-dione (DNQX, 20 μM), with the use of gramicidin-perforated recording technique which preserves the [Cl^−^]_i_ of the recorded cell [30]. The GABAergic PSPs were inhibitory in all MNCs studied in 13 virgin (n = 38 cells; Fig. 2a, *lower panel*) and 8 Dry3 rats (n = 22 cells), and in 28 of 30 cells examined in 12 Lac1 rats (Fig. 2b). On the other hand, they were excitatory in 37 of 56 MNCs recorded in 16 Lac3 rats (Fig. 2a, *upper panel*; b), and in 2 of 30 cells from Lac1 rats. The excitatory PSPs were blocked by the GABA_A_ receptor antagonist bicuculline (30 μM; n = 5; example trace not shown) and mimicked by the GABA_A_ receptor agonist muscimol (10 μM, 10 ms; n = 12; Fig. 2c). Since GABAergic excitation occurs virtually in all of the MNCs of rats subjected to chronic hyperosmotic stress [12], we checked the plasma osmolality of Lac3 rats and found it to be in the normal range (310 ± 3.0 mOsm/kg H_2_O, n = 5). Collectively, these data indicate that GABA_A_ receptor-mediated inhibitory synaptic transmission is converted into excitatory one in most MNCs of the multiparous lactating rats, but a similar change in GABAergic transmission does not occur in primiparous animals. In addition, they suggest that the GABAergic excitation in Lac3 rats does not result from osmotic stress, and that it is reversed to inhibition after the end of lactation.

### A large depolarizing E_GABA_ shift exceeding the action potential threshold was responsible for the emergence of GABAergic excitation in the MNCs of the rats in third lactation

**Fig. 3:**
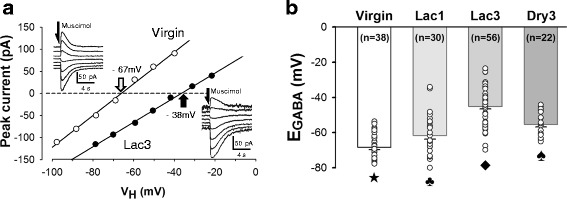
Depolarizing shift of E_GABA_ in the MNCs of multiparous lactating rats. (**a**) Estimation of E_GABA_ with the use of the currents elicited by focally applied muscimol (10 μM, 10 ms) at various holding potentials (V_H_) in the MNCs of virgin (inset; *upper left*) and Lac3 rats (inset; *lower right*). These current traces were obtained after the blockade of fast sodium current and glutamatergic transmission with the cocktail of tetrodotoxin (0.5 μM), AP-5 (100 μM) and DNQX (20 μM). Peak amplitudes of the muscimol-elicited currents are plotted against V_H_. Linear regression was used to fit the data points. The intersections (,) of the regression lines with the abscissa were taken as the reversal potentials of the muscimol-elicited responses (i.e., E_GABA_’s). (**b**) Dot plots and bar graphs showing the ranges and means (±SEM) of the E_GABA_’s of the MNCs of Virgin, Lac1, Lac3 and Dry3 rat groups. Holm-Sidak all pairwise comparison tests performed after one-way ANOVA (*p* < 0.001) indicated that these rat groups were different from one another in the mean E_GABA_. This was denoted with different symbols associated with the bar graphs. n: the number of cells examined

In order to identify the neurophysiological basis for the emergence of GABAergic excitation in the MNCs of Lac3 rats, we next estimated the E_GABA_ in these neurons, with the use of the currents elicited at various holding potentials by focal application of muscimol (10 μM, 10 ms) in the presence of tetrodotoxin (0.5 μM) (Fig. 3a). As expected, the E_GABA_ was found to be positive to the action potential threshold (which was about −45 mV) in most of the MNCs of Lac3 rats (37 of 56 cells from 16 rats); the E_GABA_ of these cells was −45.1 ± 1.4 mV (n = 56), which was significantly less negative than the E_GABA_’s of the MNCs of virgin (−68.4 ± 1.2 mV; n = 38 neurons from 13 rats), Lac1 (−61.7 ± 2.0 mV; n = 30 neurons from 12 rats) and Dry3 rats (−55.4 ± 1.4 mV; n = 22 neurons from 8 rats) (Fig. 3b). Interestingly, E_GABA_ was significantly less negative in the MNCs of Lac1 and Dry3, than virgin, groups of rats, and in the cells of Dry3, than Lac1, group (Fig. 3b). Taken together, these results indicate that a large depolarizing shift of E_GABA_ surpassing the action potential threshold is responsible for the emergence of GABAergic excitation in the MNCs of multiparous lactating rats. In addition, they suggest that GABAergic inhibition is weakened in the MNCs of Lac1 rats and, to a greater extent, in the cells of Dry3 animals.

### GABAergic excitation occurred in both the AVP and oxytocin neurons of multiparous lactating rats

**Fig. 4:**
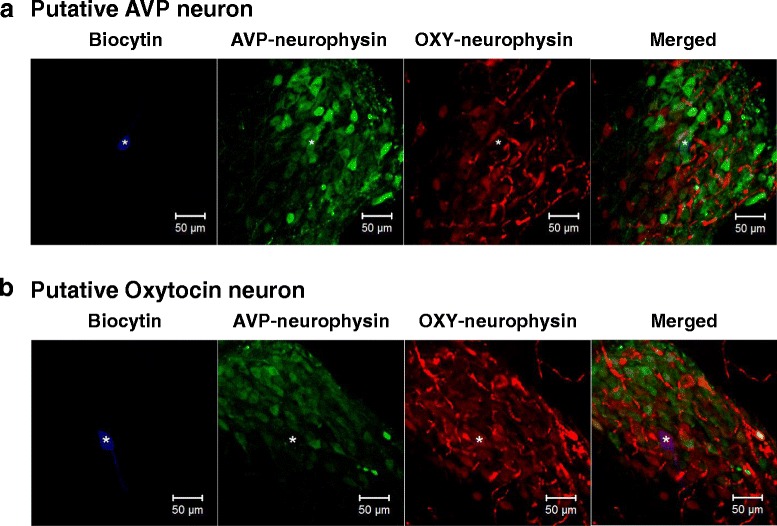
IHC identification of MNCs recorded in SON slices. (**a**, **b**) Double IHC staining for AVP-neurophysin and oxytocin-neurophysin in the MNCs of Lac3 rats injected with biocytin at the end of the recording. The biocytin-labeled cell expresses AVP-neurophysin in (**a**), whereas OXY-neurophysin in (**b**). Asterisks indicate recorded cells

To see whether the MNCs showing GABAergic excitation in Lac3 rats were AVP or oxytocin neurons, we performed double immunohistochemistry (IHC) for AVP and oxytocin neurophysin in 16 MNCs recorded in SON slices from 8 Lac3 rats. The recorded cells were marked with the use of biocytin in the internal solution. Our IHC analyses showed that the biocytin-labeled cells were positive for AVP (n = 11 cells) or oxytocin neurophysin (n = 5 cells) (Fig. 4), indicating that GABAergic excitation occurs in both AVP and oxytocin neurons.

### Depolarizing shift of E_GABA_ and the resultant emergence of GABAergic excitation were due to the up-regulation of Na^+^-K^+^-2Cl^−^ cotransporter isotype 1 (NKCC1) and down-regulation of K^+^-Cl^−^ cotransporter isotype 2 (KCC2)

**Fig. 5:**
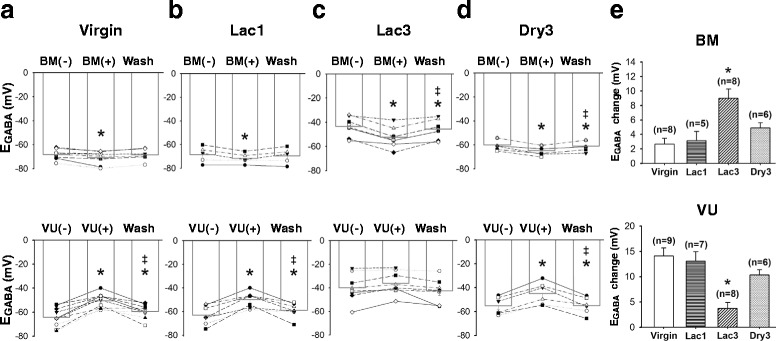
The up-regulation of NKCC1 and the down-regulation of KCC2 are responsible for the depolarizing shift of E_GABA_. (**a-d**) Graphs showing the impact of NKCC inhibitor bumetanide (BM; 10 μM) and the KCC2 blocker VU0463271 (VU; 5 μM) on the E_GABA_ of the MNCs of the Virgin, Lac1, Lac3 and Dry3 groups of rats. The symbols connected by lines denote data from the same cells. Holm-Sidak all pairwise comparison tests were performed after one-way repeated measures ANOVA (*p* = 0.001-0.04). Asterisks indicate significant difference from the data obtained before the drug application, while double daggers denote significant difference from the data obtained during the drug application. (**e**) Bar charts summarizing the hyperpolarizing effects of bumetanide and the depolarizing effects of VU0463271 on the E_GABA_ of the MNCs of different groups of rats. Holm-Sidak all pairwise comparison tests were performed after one-way ANOVA (*p* < 0.001). Asterisk indicates significant difference of the value of Lac3 group from those of other groups

It is generally agreed that the [Cl^−^]_i_ is a major determinant for E_GABA_ and the polarity and strength of GABA_A_ receptor-mediated PSP, and that NKCC1 and KCC2 play a pivotal role for Cl^−^ homeostasis in CNS neurons [6]. In the next set of experiments, we examined whether and how these co-transporters contributed to the depolarizing shift of E_GABA_ in the MNCs of Lac3 rats, by comparing virgin, Lac1, Lac3 and Dry3 rats in terms of the effects of the NKCC inhibitor bumetanide and the selective KCC2 inhibitor VU0463271 on the E_GABA_. Bumetanide (10 μM) and VU0463271 (5 μM) reversibly hyperpolarized and depolarized the E_GABA_, respectively, in all groups of rats (Figs. 5a-d). However, the effects of these drugs were not uniform in magnitude across different rat groups; while bumetanide hyperpolarized the E_GABA_ most in the Lac3 group (Fig. 5e, *upper panel*), VU0463271 depolarized the E_GABA_ to a greater extent in the Virgin, Lac1 and Dry3, than Lac3, groups (Fig. 5e, *lower panel*). The Virgin, Lac1 and Dry3 groups were not significantly different from one another with regard to the magnitudes of the bumetanide and VU0463271 effects (Fig. 5e). Thus, these results collectively suggested that the depolarizing shift of E_GABA_ in the MNCs of Lac3 rats arises from the combination of NKCC1 up-regulation and KCC2 down-regulation.

## Discussion

In the current study we examined GABA_A_ receptor-mediated transmission in rat MNCs. We found that, during the period of lactation, the E_GABA_’s of MNCs depolarize significantly such that the GABAergic inhibition of these cells was weakened in primiparous animals while mostly converted into excitation in multiparous females. Furthermore, we obtained evidence that the inhibitory-to-excitatory switch in GABAergic transmission is reversed after the cessation of lactation. Thus, the results of the current study indicate that the GABAergic responses of rat MNCs are reversibly modulated during lactation by the depolarizing shift of E_GABA_ and that this plastic change is more marked in animals with more reproductive experiences.

The switch from GABA inhibition to excitation in the MNCs would be expected to increase secretion of oxytocin and AVP in lactating females. Brussaard and his co-workers [31, 32] have found that, around parturition and during the period of lactation in the rat, the inhibitory effect of GABA on oxytocin neurons is attenuated by the postsynaptic alteration of GABA_A_ receptor subunit composition and the consequent removal of the potentiating effects of neurosteroids on the function of this receptor. Thus, they postulated that the reduction of GABAergic restraint on oxytocin neurons underlies the increased oxytocin secretion at particular times of the reproductive cycle in the rat. On the other hand, Moos [33] has reported that, although GABA suppresses the baseline electrical activities of oxytocin neurons, it paradoxically facilitates the suckling-induced bursting activities of these cells to enhance their outputs. These interpretations will need to be re-examined in light of our observations of GABA-mediated excitation in the experienced lactating females.

The present study demonstrates that the E_GABA_ is significantly less negative in the cells of Dry3, than virgin and Lac1 rats. These findings indicate that the depolarizing shift of E_GABA_ in multiparous lactating rats is not fully reversed after the end of lactation although the E_GABA_ gets repolarized enough to prevent the occurrence of GABAergic excitation. We do not know why, after the end of lactation, the E_GABA_ does not return to baseline levels. It is possible that the GABA excitation would fully reverse in time. We speculate that less negative E_GABA_ in multiparous dry rats may help to speed up the change in GABAergic transmission that has to occur in the next lactation and that this plasticity may be a salient feature of the brain of the rat with multiple reproductive experiences [34, 35].

The transmembrane Cl^−^ gradient is a critical factor determining the strength and the polarity of GABA_A_ receptor-mediated synaptic response. In CNS neurons, [Cl^−^]_i_ is regulated by the Cl^−^ importer NKCC1 and the Cl^−^ extruder KCC2 [36]. In this study, we provided neurophysiological evidence that the up-regulation of NKCC1 and the down-regulation of KCC2 are responsible for the depolarizing shift of E_GABA_ and the resultant emergence of GABAergic excitation in the MNCs of multiparous lactating rats. However, we did not identify the mechanisms linking lactation to the changes in NKCC1 and KCC2. It is well established that AVP and oxytocin are released in the SON and PVN and this local release is enhanced during the period of lactation [37] when AVP can act as a paracrine signal to induce inward current in oxytocin neurons [38]. In addition, we have previously shown that the intracerebroventricular administration of selective oxytocin receptor antagonist partially obstructs the depolarizing E_GABA_ shift, which is induced by NKCC1 up-regulation, and the consequent emergence of GABAergic excitation in the MNCs of rats subjected to chronic hyperosmotic stress [12]. Meanwhile, a recent study reported that, in a rat model of hypertension produced by salt loading, brain-derived neurotrophic factor (BDNF)-tropomyosin-receptor-kinase B (TrkB) activation causes the down-regulation of KCC2 and the depolarizing shift of E_GABA_ in MNCs [39]. Thus, it is possible that the somato-dendritic release of oxytocin and the BDNF-TrkB activation are the mechanisms underlying the NKCC1 up-regulation and KCC2 down-regulation in the MNCs of multiparous rats.

## Conclusion

In the mammal, the secretion of oxytocin and AVP from MNCs is increased during lactation. In the current study we found that the E_GABA_’s of MNCs depolarize during the period of lactation such that the GABAergic inhibition of these cells was weakened in primiparous rats and mostly converted into excitation in multiparous females. Furthermore, we obtained evidence that the inhibitory-to-excitatory switch in GABAergic transmission, which is driven by a combination of NKCC1 up-regulation and KCC2 down-regulation, is reversed after the cessation of lactation. We conclude that the GABAergic responses of rat MNCs are modulated reversibly during lactation, perhaps to enhance the secretion of AVP and oxytocin.

## Materials and methods

### Animal care

Female Sprague–Dawley rats (250–400 g) from Orient Bio Co (Sungnam, Korea) were used in the current study. They were housed in a temperature-controlled vivarium (22-24 °C) with a 12/12-h light/dark cycle. The experimental procedures described below were approved by the Institutional Animal Care and Use Committee and conformed to the guidelines of the National Institutes of Health Guide for the Care and Use of Laboratory Animals. All possible efforts were made to minimize the number of animals used as well as their suffering.

### Animal groups

Four groups of rats were used in this study: virgin rats (Virgin), primiparous lactating rats (Lac1) or rats in third lactation (Lac3) and rats in dry period after giving birth 3 times (Dry3). Virgin and Dry3 groups of rats were killed for hypothalamic slices in ≥7 days after their arrival at the vivarium, while Lac1 and Lac3 groups of rats were sacrificed after giving births to and then nursing their pups (n = 8-12) for 3–14 days in the vivarium. Dry3 rats had not been pregnant for 1–3 months after the end of last lactation.

### Hypothalamic slice preparation

Hypothalamic slices were prepared as previously described [40]. In brief, the rat was anesthetized with urethane (1.25 g/kg, i.p.), and the brain was quickly excised from the skull and submerged in ice-cold artificial cerebrospinal fluid (ACSF; composition in mM: 124 NaCl, 1.3 MgSO_4_, 3 KCl, 1.25 NaH_2_PO_4_, 26 NaHCO_3_, 2.4 CaCl_2_, and 10 glucose). After being chilled for 1–2 min, the brain was trimmed to a block containing the hypothalamus. Using a vibroslicer (Campden Instruments, Loughborough, United Kingdom), coronal slices (350 μm) containing the SON were cut from the tissue block in ice-cold ACSF. The slices were transferred to a gas-interface type recording chamber, which was perfused with warm (34-35 °C) aerated (95 % O_2_/5 % CO_2_) ACSF at a rate of 0.5-1 mL/min by a peristaltic pump-driven or gravity-fed bath perfusion system [9]. Warm (34-35 °C) air humidified by 95 % O_2_/5 % CO_2_ gas mixture was continuously blown over the slices to further ensure adequate oxygenation of cells in the tissue.

### Intracellular electrophysiological recording

Current or voltage clamp recordings were obtained from neurons in the SON of hypothalamic slices equilibrated for 1–8 h in the recording chamber as described previously [12]. In brief, the SON was identified as a translucent region right next to the optic chiasm. Micropipettes (tip diameter, 1.5-2.0 μm; 3–5 MΩ) pulled from borosilicate glass capillaries (P-97; Sutter Instrument Co, Novato, CA) and filled with gramicidin (50 μg/mL)-containing solution (composition in mM: 143 K-gluconate, 2 KCl, 10 HEPES, and 0.5 EGTA; pH 7.2-7.3) were used for recording in a perforated configuration. Stable perforated recording condition was usually achieved 10–25 min after seal was formed. Those recordings having steady series resistances (range 30–50 MΩ) and action potential amplitudes of >45 mV (measured from action potential threshold) were the only ones included in the data pool. The voltage errors resulting from the series resistance were compensated offline for voltage clamp recordings and online for current clamp recordings by using the bridge circuit. We corrected the liquid junction potential before the experiments; we set the pipette potential to −9 mV just before the formation of patch configuration, knowing that the liquid junction potential was 15.8 mV (at 34.5 °C) while the perforated patch potential arising from gramicidin perforation was −6.8 mV. We assumed that the change in resting membrane potential detected when the recording mode was transformed from perforated to whole-cell configuration represented the perforated patch potential. The signals from neurons amplified by Axoclamp-2B amplifier (bandwidth filter set at 10 Hz) were digitized and sampled at 50 μs intervals (Digidata1320, pClamp 8.0; Molecular Devices, Sunnyvale, CA).

### Drugs

We purchased all the drugs and chemicals used in the current study from Sigma-Aldrich, except for muscimol (GABA_A_ receptor agonist; Ascent Scientific, Cambridge, MA) and VU0463271 (KCC2 blocker; gift from Prof. Craig Lindsley, Vanderbilt University in Nashville, TN, USA). We prepared the solutions of muscimol and AP-5 (NMDA receptor antagonist) by dissolving these drugs in ACSF, the standard slice perfusion medium, and DNQX (non-NMDA receptor antagonist) solution by diluting its dimethylsulfoxide-based stock solution with ACSF (final concentrations of dimethylsulfoxide, 0.05 %). The solution of bumetanide (NKCC blocker) and VU0463271 was prepared by dissolving this agent in AP-5 and DNQX containing ACSF. Muscimol solution was applied focally by “Y-tube” method [41], while other drug solutions by bath perfusion.

### IHC identification of recorded neurons

For post-hoc IHC identification of SON neurons recorded in slices from the Lac3 rats, the cells were infused with biocytin; biocytin (1.5 mg/mL) contained in the recording pipette was allowed to get into the cell by holding the cell in whole-cell mode for 10–15 min after perforated patch recordings. The SON slices were fixed in 4 % paraformaldehyde-containing phosphate buffered saline (PBS, 0.1 M) at pH 7.4 for 24 h, transferred to 30 % sucrose-PBS and kept in this solution for 24–48 h. Then, they were cut into 25–35 μm-thick sections. These sections were incubated for 24 h at 4 °C in a solution containing a rabbit polyclonal antibody against AVP-neurophysin (1:200; Abcam, Cambridge, MA) and a mouse monoclonal antibody against oxytocin-neurophysin (1:5000; Abcam). After being washed with PBS three times, the sections were reacted with DyLight 488-conjugated goat anti-rabbit and DyLight 594-conjugated goat anti-mouse secondary antibodies (1:200 dilution each; Jacson ImmunoResearch, West Grove, PA) for 24 h at 4 °C, and then with avidinAMCA (1:500 dilution; Vector Labs, Burlingame, CA) for 1 h at room temperature. The secondary antibodies and avidinAMCA were dissolved in 0.1 M PBS containing 0.3 % Triton X-100 and 2 % normal goat serum. The sections were examined under a confocal fluorescence microscope for the presence of AVP-neurophysin and oxytocin-neurophysin immunoreactivity and biocytin labeling.

### Statistical analysis

Numerical data are expressed as the mean ± SEM. Student’s *t* test was used for the comparison of two independent datasets with normal distribution. One-way analysis of variance (ANOVA) and one-way repeated measures ANOVA were performed to compare multiple independent and dependent datasets with normal distributions, respectively. The pairwise comparisons following the ANOVA’s were done with Holm-Sidak method. Chi-square test was performed to determine whether there is a significant difference between the observed frequencies in one or more categories. *P* < 0.05 was considered significant. This significance level, however, was reduced with the Bonferroni correction when the problem of multiple comparisons arises.

## Abbreviations

ACSF: Artificial cerebrospinal fluid
ANOVA: One-way analysis of variance
AP-5: DL-2-amino-5-phosphonopentanoic acid
AVP: Arginine vasopressin
BDNF: Brain-derived neurotrophic factor
DNQX: 6,7-dinitroquinoxaline-2,3-dione
E_GABA_: Reversal potential of GABA_A_ receptor-mediated response
IHC: Immunohistochemistry
KCC2: K^+^-Cl^−^ cotransporter isotype 2
MNCs: Magnocellular neurosecretory cells
NKCC1: Na^+^-K^+^-2Cl^−^ cotransporter isotype 1
PBS: Phosphate buffered saline
PND: Postnatal day
PVN: Paraventricular nucleus
SON: Supraoptic nucleus
TrkB: Tropomyosin-receptor-kinase B
